# Applicability of Creatinine-based equations for estimating glomerular filtration rate in elderly Chinese patients

**DOI:** 10.1186/s12877-021-02428-y

**Published:** 2021-09-04

**Authors:** Fangxiao Xia, Wenke Hao, Jinxiu Liang, Yanhua Wu, Feng Yu, Wenxue Hu, Zhi Zhao, Wei Liu

**Affiliations:** 1 Department of Nephrology, Guangdong Provincial People’s Hospital, Guangdong Academy of Medical Sciences, Guangdong Provincial Geriatrics Institute, Guangzhou, 510080 China; 2 The Second School of Clinical Medicine, Southern Medical University, Guangzhou, 510515 China

**Keywords:** Elderly, Estimated GFR, Serum creatinine, Equation

## Abstract

**Background:**

The accuracy of the estimated glomerular filter rate (eGFR) in elderly patients is debatable. In 2020, a new creatinine-based equation by European Kidney Function Consortium (EKFC) was applied to all age groups. The objective of this study was to assess the appropriateness of the new EKFC equation with Chronic Kidney Disease Epidemiology Collaboration (CKD-EPI), Lund-Malmö Revised (LMR), Berlin Initiative Study 1 (BIS1), and full age spectrum (FAS) equations based on serum creatinine (SCR) for elderly Chinese patients.

**Methods:**

A total of 612 elderly patients with a measured glomerular filtration rate (mGFR) by the dual plasma sample clearance method with Technetium-99 m-diethylenetriamine-pentaacetic acid (Tc-99 m-DTPA) were divided into four subgroups based on age, sex, mGFR, and whether combined with diabetes. The performance of GFR was assessed while considering bias, precision, accuracy, and root-mean-square error (RMSE). Bland-Altman plots, concordance correlation coefficients (CCCs), and correlation coefficients were applied to evaluate the validity of eGFR.

**Results:**

The median age of the 612 participants was 73 years, and 386 (63.1%) were male. Referring to mGFR (42.1 ml/min/1.73 m^2^), the CKD-EPI, LMR, BIS1, FAS, and EKFC equations estimated GFR at 44.4, 41.1, 43.6, 41.8 and 41.9 ml/min/1.73 m^2^, respectively. Overall, the smallest bias was found for the BIS1 equation (− 0.050 vs. range − 3.015 to 0.795, *P*<0.05, vs. the CKD-EPI equation). Regarding P30, interquartile range (IQR), RMSE, and GFR category misclassification, the BIS1 equation generally performed more accurately than the other eqs. (73.9%, 12.7, 12.9, and 35.3%, respectively). Nevertheless, no equation achieved optimal performance for the mGFR≥60 ml/min/1.73 m^2^ subgroup. Bland-Altman analysis showed the smallest mean difference (− 0.3 ml/min/1.73 m^2^) for the BIS1 equation when compared to the other equations.

**Conclusions:**

This study suggested that the BIS1 equation was the most applicable for estimating GFR in Chinese elderly patients with moderate to severe renal impairment.

## Background

Chronic kidney disease (CKD) is considered a global public health problem. It has been reported that by 2017, the global prevalence of CKD was 9.1% and that approximately 1.2 million people had died of CKD; of these, the number of patients with kidney disease in China was approximately 1.323 billion, reaching 9.5 %[1]. CKD is common in elderly individuals, who account for an increasing proportion of the total population [2]. This is not only the result of the physiological ageing of the kidney but also the result of the impact of certain diseases [2]. Thus, for patients over 65 years old, especially those with CKD, accurate measurement of GFR is particularly important for diagnosis and treatment as well as evaluating the prognosis of elderly patients.

To date, the referred methods for obtaining the measured glomerular filtration rate (mGFR) have included clearance of inulin, iohexol, ^51^Cr-EDTA, and Tc-99 m-DTPA, which accurately assess kidney function and significantly reduce errors produced by variables (such as sex, age, and race) in the eGFR equations. Nevertheless, determining mGFR is relatively complicated and not feasible in daily clinical practice. Kidney Disease: Improving Global Outcomes (KDIGO) guidelines recommend using GFR-estimating equations as noninvasive alternatives [3].

Through comparison of the Cockcroft-Gault equation [4] and modification of diet in renal disease (MDRD) equation [5] to the chronic kidney disease epidemiological collaboration (CKD-EPI) equation [6, 7], researchers had reported that the CKD-EPI equation, which had been recommended by guidelines, was more accurate [3, 8]. Although eGFR equations had been greatly improved, they were not developed in elderly individuals. Several new eGFR equations for the elderly population have been reported. In 2012, the Berlin Initiative Study (BIS) used the iohexol plasma clearance method as a reference [9] to derive the BIS equations for white individuals over 70 years old. Pottel et al. in 2016 developed the full age spectrum (FAS) equation to evaluate GFR based on a study of European healthy subjects [10]. A recent study showed that there was no better diagnostic performance for 65 years and older who had GFR estimated using CKD-EPI, BIS1, LMR, and FAS equation-based SCR [11]. In 2020, the European Renal Function Alliance developed and verified a new equation based on SCR by combining the design performance of the FAS and CKD-EPI equations. This equation can be applied to all age groups, as can the FAS equation. Moreover, the new EKFC equation showed higher accuracy and precision than commonly used equations such as CKD-EPI [12]. However, the new equation was developed using white individuals as the research subjects, and it has not been verified whether it is suitable for the Chinese elderly population. Therefore, this study was conducted to evaluate the performance of five equations based on SCR: CKD-EPI, LMR, FAS, BIS1, and EKFC.

## Methods

### Study population and setting

**Fig. 1:**
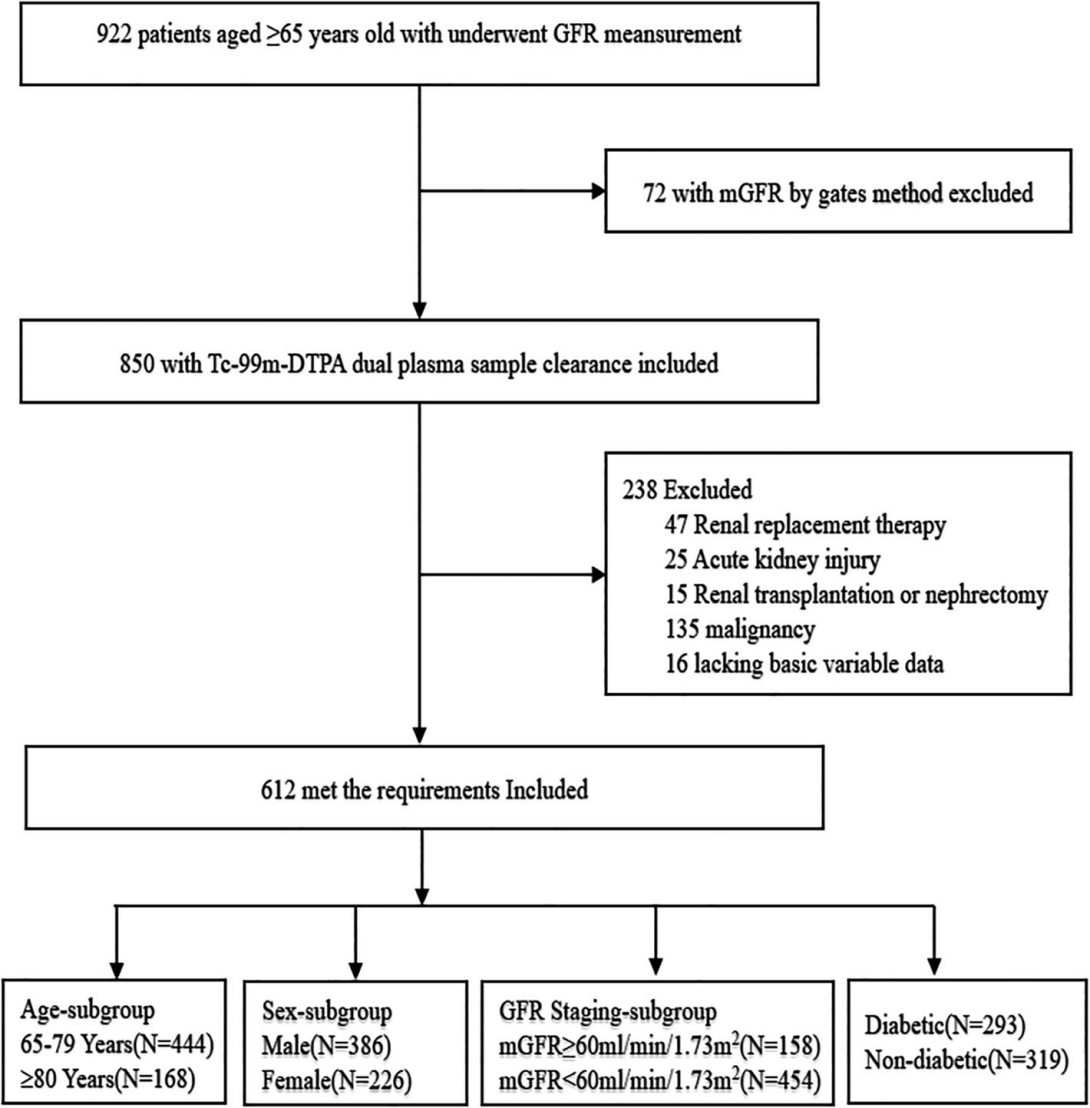
Flowchart of the study

This retrospective study was carried out to include all consecutive patients 65 years and older who underwent GFR measurement by the Tc-99 m-DTPA dual plasma sample clearance method between January 1, 2010, and December 31, 2019. A total of 612 inpatients were enrolled from the medical wards of Guangdong Provincial People’s Hospital. Exclusion criteria are shown in Fig. 1. The diagnostic criteria for CKD referred to the KDIGO: clinical evidence of renal damage> 3 months or GFR < 60 ml/min/1.73 m^2^ for 3 months or more [3].

### Measurement of GFR

The mGFR value was assessed using the Tc-99 m-DTPA dual plasma sample clearance method. Three millilitres of venous blood was collected from the elbow vein of subjects at 2 h and 4 h after the injection of Tc-99 m-DTPA. Venous blood was anticoagulated with heparin and centrifuged at 2000 r/min. Then, 1 ml plasma was withdrawn and placed on a radioimmune γ counter to determine the radioactivity count for 60 s. Each sample was measured three times; the highest and lowest counts were removed, and the middle count was used into the following formula:

$$ \mathrm{GFR}=\left[\mathrm{Dln}\left(\mathrm{P}1/\mathrm{P}2\right)/\left(\mathrm{T}2-\mathrm{T}1\right)\right]\exp \left[\left(\mathrm{T}1\ \mathrm{lnP}2\right)-\left(\mathrm{T}2\ \mathrm{lnP}1\right)\right]/\left(\mathrm{T}2-\mathrm{T}1\right)\times 1.73/\mathrm{BSA} $$

where D is the radioactivity count of the drug injected into the body; T1 is the first blood collection time (120 min); P1 is the radioactivity count in plasma at T1; T2 is the second blood collection time (240 min); P2 is the radioactivity count in plasma at T2; and body surface area (BSA) = height(cm)^0.725^ × weight(kg)^0.425^ × 0.007184 mGFR was employed as the reference.

### Determination of serum creatinine

Blood samples were collected from each participant before the dual plasma sample clearance method and analysed in the same laboratory at Guangdong Provincial People’s Hospital. SCR was measured using the picric acid method and an autoanalyzer (Beckman Coulter AU5800, America) with a reference range of 57–110 μmol/L for males and 53–97 μmol/L for females. All plasma creatinine levels were measured with methods traceable to the National Institute of Standards and Technology (isotope-dilution mass spectrometry calibrated) and creatinine standard reference material (SRM 909b).

### GFR-estimated equations

**Fig. 2:**
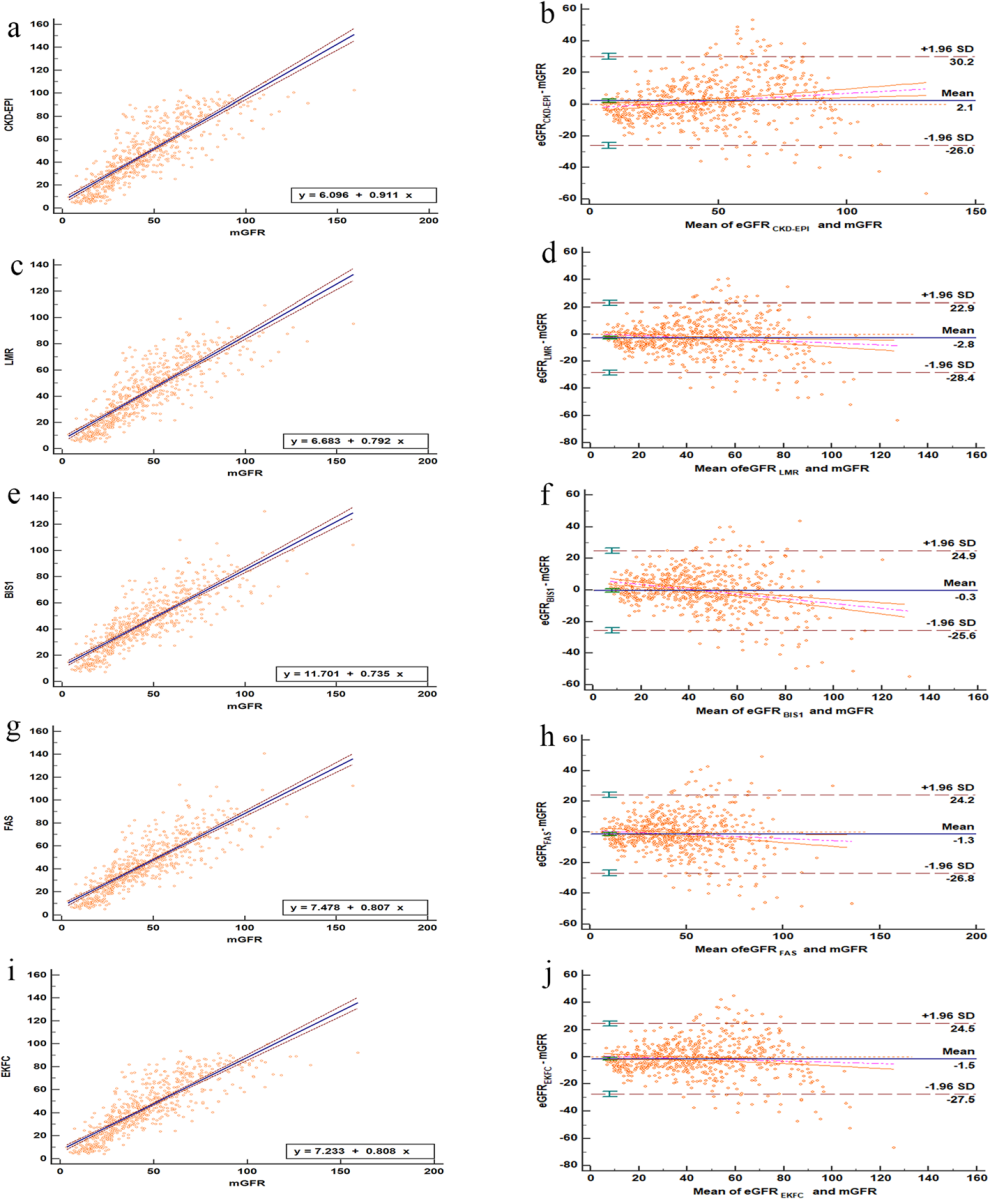
Comparisons eGFR and mGFR. **a**, **b** CKD-EPI equation; **c**, **d** LMR equation; **e**, **f** BIS1 equation. **g**, **h** FAS equation; **i**, **j** EKFC. Solid and dashed lines in the Bland-Altman plot represent the mean and 95% limits of agreement (LoA) of difference, respectively. Abbreviations: *CKD-EPI*, Chronic Kidney Disease Epidemiology; *LMR*, Lund-Malmö Revised; *BIS1*, Berlin Initiative Study 1; *FAS*, full age spectrum; *EKFC*, European Kidney Function Consortium

The equations used in this study were presented in Table 1. Scatter plots and Bland–Altman plots of the five equations versus mGFR were depicted in Fig. 2.

**Table 1:** The expression of five equations basing on SCr in the study

Year	Equation Name	Equation
2009	CKD- EPI equation	141 × (SCr/0.9)^−0.411^ × 0. 993^Age^(Male, SCr ≤ 0.9)
		141 × (SCr/0.9)^−1.209^ × 0. 993^Age^(Male, SCr > 0.9)
		144 × (SCr/0.7)^−0.329^ × 0. 993^Age^(Female, SCr ≤ 0.7)
		144 × (SCr/0.7)^−1.209^ × 0. 993^Age^(Female, SCr > 0.7)
2011	LMR equation	e^X – 0.0158 ∗ Age + 0.438 ∗ In(Age)^
		X = 2.56 + 0.00968 ∗ (2 – SCr)(Male, Scr < 2.0)
		X = 2.56 – 0.926 ∗ In(SCr/2) (Male, Scr ≥ 2.0)
		X = 2.50 + 0.0121 ∗ (1.7 – SCr) (Female, SCr < 1.7)
		X = 2.50 – 0.926 ∗ In (SCr/1.7) (Female, SCr ≥ 1.7)
2012	BIS1 equation	3736 × SCr^−0.870^ × age^−0.950^ × (0.82 Female)
2016	FAS equation	107.3/(SCr/0.9) × [0. 988^(age − 40)^age > 40 years](Male)
		107.3/(SCr/0.7) × [0. 988^(age − 40)^age > 40 years](Female)
2020	EKFC equation	107.3 × (SCr/0.9)^−0.322^ × [0.990^(age − 40)^age > 40 years] (Male, SCr < 0.9)
		107.3 × (SCr/0.9)^−1.132^ × [0.990^(age − 40)^age > 40 years] (Male, SCr ≥ 0.9)
		107.3 × (SCr/0.7)^−0.322^ × [0.990^(age − 40)^age > 40 years](Female, SCr < 0.7)
		107.3 × (SCr/0.7)^−1.132^ × [0.990^(age − 40)^age > 40 years] (Female, SCr ≥ 0.7)

*Abbreviations*: *Scr (mg/dl)* Serum creatinine, *CKD-EPI* Chronic kidney disease epidemiology collaboration, *LMR* Lund-Malmö Revised, *BIS1* Berlin Initiative Study 1, *FAS* Full age spectrum equation, *EKFC* European Kidney Function Consortium. serum creatinine expressed as mg/dl while 1 mg/dl equal to 88.4 μmol/l

### Statistical analysis

Data analysis was conducted using IBM SPSS Software (version 26. 0 SPSS, IBM Corp) and Medcalc for Windows (version 19. 1 Medcalc Software, Mariekerke, Belgium). The study assessed the performance of five equations with metrics of bias (median difference between eGFR and mGFR), precision (interquartile range of the median difference [IQR]), and accuracy (the proportion of eGFR within 30% of mGFR [P30] and the root-mean-square error [RMSE] ($\sqrt{\frac{\varSigma_i^n{\left( eGFRi- mGFRi\right)}^2}{n}}$) of eGFR calculated by the five equations), as suggested by the NKF/KDOQI guideline [13], and the GFR category misclassification rate. Data concerning mGFR and eGFR did not follow a normal distribution (Kolmogorov-Smirnov test, *P* < 0.05) and were analysed by nonparametric tests. Negative bias indicated that an equation underestimated GFR and vice versa. Higher P30 or smaller RMSE implied better accuracy. Mean absolute error (MAE) denoted the mean of the absolute error between eGFR and mGFR values, similar to RMSE. The GFR category misclassification rate was calculated as the proportion of participants predicted to be at an incorrect stage using eGFR. The concordance correlation coefficient (CCC) was applied to assess the strength of the theoretical agreement between each eGFR and mGFR. Spearman correlation analysis was used to compare the correlation between eGFR and mGFR with each equation. Cohen’s kappa (κ) was employed to quantify agreement between eGFR and mGFR in identifying people with different degrees of renal impairment. The area under the receiver operator characteristic (ROC) curve (AUC) was used to determine the ability of eGFR equations to discriminate between elderly patients with and without CKD. The Youden index also reflected the authenticity of the eGFR equation, and a larger value showed better authenticity. Bland–Altman plot was used to calculate the mean difference and precision between eGFR and mGFR. The Wilcoxon matched-pairs signed-rank test and McNemar’s test were implemented to compare bias and accuracy, respectively. *P* < 0.05 was considered statistically significant.

## Results

### Participant characteristics

Altogether, 612 participants with a median age of 73 (68, 80) years old were enrolled in this study, including 386 (63.1%) males. The participants were divided into different subgroups by sex, age, GFR staging, and presence of diabetes. The median SCR (μmol/l) was 119.62 overall, 132.60 in the male group, and 106.50 in the female group. The median mGFR (ml/min/1.73 m^2^) was 42.1 overall, 43.7 in the male group, and 40.1 in the female group. The median eGFR (ml/min/1.73 m^2^) by the different equations ranged from 41.1 to 44.4 overall, from 40.9 to 44.8 in the male group, and from 40.8 to 44.3 in the female group. Approximately 75.2% of the subjects had hypertension, and 47.9% had diabetes. The detailed demographic and clinical characteristics of the participants were listed in Table 2**.**

**Table 2:** Characteristics of participants

Characteristics	Whole Cohort	Male	Female	***P***
Participants	612	386	226	
Age (Years)	73 (68,80)	75 (69,82)	72 (68,77)	0.000^*^
BSA(m^2^)	1.61 (1.48,1.74)	1.69 (1.56,1.81)	1.52 (1.42,1.52)	0.000^*^
Scr (μmol/l)	119.62 (86.00,190.80)	132.60 (89.60,183.71)	106.50 (74.0,205.8)	0.001^*^
Bun (mmol/l)	8.18 (6.10,12.18)	8.10(6.17,11.52)	8.26 (6.00,13.13)	0.927^*^
Alb(g/l)	34.80 (30.70,38.30)	34.60 (30.70,38.00)	35.10 (30.38,39.00)	0.439^*^
Year group, n(%)				
65–79 Year	444 (70.13)	256 (65.20)	188 (79.36)	0.000**
≥80	168 (29.87)	130 (34.80)	38 (20.64)	
mGFR (ml/min/1.73 m^2^)	42.1 (27.4,62.1)	43.7 (27.9,61.2)	40.1 (25.6,59.2)	0.080^*^
mGFR, n(%)				
≥ 90 ml/min/1.73 m^2^	32 (5.2)	21 (5.4)	11 (4.9)	
60–89 ml/min/1.73 m^2^	126 (20.6)	82 (21.2)	44 (19.5)	
30–59 ml/min/1.73 m^2^	266 (43.5)	175 (45.3)	91 (40.3)	
15–29 ml/min/1.73 m^2^	146 (23.9)	90 (23.3)	56 (24.8)	
< 15 ml/min/1.73 m^2^	42 (6.9)	18 (4.7)	24 (10.6)	
eGFR (ml/min/1.73 m^2^)				
CKD-EPI equation	44.4 (27.1,67.0)	44.8 (29.3,65.8)	43.8 (20.0,69.4)	0.390^*^
LMR equation	41.1 (23.9,59.9)	40.9 (25.0,60.0)	41.5 (18.8,63.6)	0.799^*^
BIS1 equation	43.6 (30.0,59.5)	43.4 (31.8,57.7)	44.3 (25.0,61.0)	0.778^*^
FAS equation	41.8 (27.7,59.3)	42.1 (29.6,58.8)	41.3 (21.8,60.6)	0.391^*^
EKFC equation	41.9 (26.4,61.6)	42.2 (28.1,61,1)	40.8 (19.6,64.5)	0.305^*^
Causes of kidney disease, n(%)				
Primary glomerular disease	101 (16.5)	49 (12.7)	52 (23.0)	0.000^**^
Diabetic nephropathy	164 (26.8)	105 (27.2)	59 (26.1)	0.000^**^
Hypertensive nephrosclerosis	84 (13.7)	60 (15.5)	24 (10.6)	0.000^**^
Obstructive nephropathy	100 (16.3)	49 (12.7)	51 (22.6)	0.000**
Others	163 (26.6)	123 (31.9)	40 (17.7)	0.000^**^
Comorbid condition (%)				
Hypertension	460 (75.2)	286 (74.1)	174 (77.0)	0.000^**^
Diabetes	293 (47.9)	189 (49.0)	104 (46.2)	0.000^**^
Coronary heart disease	225 (36.8)	159 (41.2)	66 (29.2)	0.000^**^
Cerebrovascular disease	133 (21.7)	103 (26.7)	30 (13.3)	0.000^**^

All data were collected from each individual in this study unless otherwise stated. Values for continuous variables are presented as the median and inter-quartile range

*Abbreviations*: *BSA* Body surface area, *Scr* Serum creatinine, *Bun* Blood urea nitrogen, *Alb* Albumin, *mGFR* Measured glomerular filtration rate, *eGFR* Estimated glomerular filtration rate, *CKD-EPI* Chronic kidney disease epidemiology collaboration, *LMR* Lund-Malmö Revised, *BIS1* Berlin Initiative Study 1, *FAS* Full age spectrum equation, *EKFC* European Kidney Function Consortium. Reference range: Scr, 57-110 μmol/l in male and 53-97 μmol/l in female; Bun, 3.60–9.50 mmol/l; Alb, 44.00–55.00 g/L.

* Non-parametric *t*-test, **Chi-square test, comparing between male and female

### Performance of different equations for all participants

**Fig. 3:**
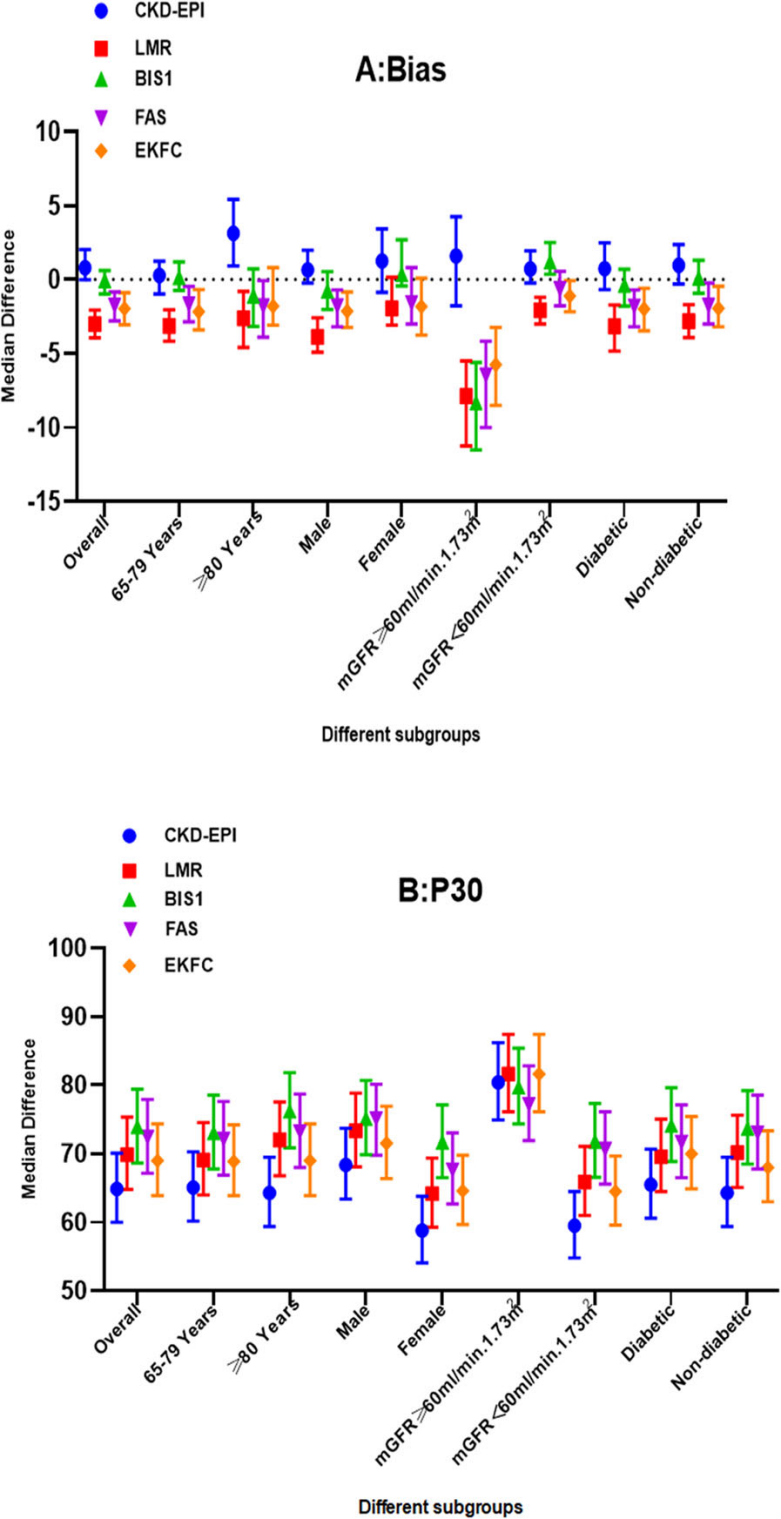
Performance of five equations for eGFR. A shows the median difference between eGFR and mGFR in different subgroups. B shows the accuracy of the ffive equations (P30). I bars indicate 95% confidence interval. CKD-EPI, Chronic Kidney Disease Epidemiology; LMR, Lund-Malmö Revised; BIS1, Berlin Initiative Study 1; FAS, full age spectrum; EKFC, European Kidney Function Consortium

In the entire cohort of participants, the Spearman correlation coefficient (Rs) of the five equations showed a significant positive correlation with mGFR, as presented in Table 3. For all subgroups, no CCC between mGFR and eGFR by any equation was greater than 0.900. In addition, the CKD-EPI equation had the lowest CCC (0.843). For the whole cohort, Cohen’s kappa of the BIS1 (*κ* = 0.485) and FAS (*κ* = 0.482) equations was slightly higher than that of the other equations. Concerning bias, all equations underestimated GFR, except for the CKD-EPI equation (Fig. 3), and the bias of BIS1 was not significant (bias: -0.050, *P* = 0.927) (Table 4). In terms of precision, IQR (ml/min/1.73 m^2^) was smallest for the BIS1 eq. (12.7), followed by the FAS eq. (13.1). The largest IQR was obtained with the CKD-EPI equation for all subgroups. The BIS1 equation was the most accurate, with the highest P30 (73.9%), whereas the CKD-EPI equation showed the lowest P30 (64.9%) (Fig. 3). The lowest overall GFR category misclassification rate was obtained with the BIS1 eq. (35.3%), followed by the FAS equation (36.3%) (Table 4). Bland-Altman analysis showed that the BIS1 equation had the smallest value (− 0.3 ml/min/1.73 m^2^) (Fig. 2**)**.

**Table 3:** Diagnostic value analysis of the five GFR-Estimating Equation

	Rs	CCC(95%CI)	ROC^**AUC**^(95%CI)	Sensitivity	Specificity	Youden index	Kappa
All Participants(*N* = 612)							
CKD-EPI	0.872^a^	0.843 (0.820,0.863)	0.925 (0.902,0.945)^*^	87.7	83.5	0.712	0.438**
LMR	0.873^a^	0.855 (0.833,0.874)	0.926 (0.903,0.946)^*^	86.6	84.8	0.714	0.473**
BIS1	0.871^a^	0.863 (0.841,0.882)	0.926 (0.902,0.945)^*^	82.4	88.6	0.710	0.485**
FAS	0.873^a^	0.864 (0.843,0.883)	0.927 (0.903,0.947)^*^	87.7	84.2	0.718	0.482**
EKFC	0.874^a^	0.849 (0.827,0.869)	0.927 (0.912,0.952)^*^	85.2	86.7	0.720	0.477**
Participants Aged 65–79 Years(*N* = 444)							
CKD-EPI	0.898^a^	0.858 (0.834,0.879)	0.943 (0.917,0.962)^*^	89.3	85.6	0.750	0.491**
LMR	0.896^a^	0.870 (0.847,0.890)	0.943 (0.917,0.962)^*^	87.2	88.8	0.760	0.478**
BIS1	0.896^a^	0.880 (0.857,0.899)	0.943 (0.917,0.962)^*^	86.8	88.8	0.756	0.489**
FAS	0.898^a^	0.880 (0.858,0.899)	0.944 (0.918,0.963)^*^	88.4	88.0	0.764	0.488**
EKFC	0.898^a^	0.863 (0.839,0.884)	0.943 (0.918,0.963)^*^	88.4	88.0	0.764	0.487**
Participants Aged ≥80 Years(*N* = 168)							
CKD-EPI	0.760^a^	0.773 (0.707,0.825)	0.858 (0.796,0.907)^*^	84.4	75.8	0.602	0.441**
LMR	0.762^a^	0.784 (0.720,0.836)	0.858 (0.796,0.907)^*^	84.4	75.8	0.602	0.438**
BIS1	0.762^a^	0.788 (0.724,0.838)	0.859 (0.797,0.908)^*^	84.4	75.8	0.602	0.453**
FAS	0.762^a^	0.791 (0.727,0.842)	0.860 (0.798,0.909)^*^	84.4	75.8	0.602	0.449**
EKFC	0.761^a^	0.784 (0.719,0.835)	0.858 (0.796,0.907)^*^	83.7	75.8	0.595	0.429**
Male(*N* = 386)							
CKD-EPI	0.862^a^	0.833 (0.801,0.860)	0.911 (0.878,0.937)^*^	85.5	84.5	0.700	0.490**
LMR	0.886^a^	0.840 (0.808,0.867)	0.913 (0.881,0.939)^*^	85.2	85.4	0.706	0.431**
BIS1	0.865^a^	0.838 (0.807,0.864)	0.914 (0.882,0.940)^*^	83.0	86.4	0.695	0.469**
FAS	0.865^a^	0.841 (0.809,0.867)	0.914 (0.881,0.940)^*^	87.3	82.5	0.698	0.464**
EKFC	0.865^a^	0.838 (0.806,0.865)	0.913 (0.880,0.939)^*^	84.8	85.4	0.702	0.442**
Female(*N* = 226)							
CKD-EPI	0.891^a^	0.852 (0.820,0.879)	0.951 (0.914,0.975)^*^	93.0	85. 5	0.784	0.467**
LMR	0.892^a^	0.872 (0.841,0.898)	0.953 (0.916,0.976)^*^	91.8	87.3	0.791	0.537**
BIS1	0.889^a^	0.894 (0.864,0.917)	0.950 (0.913,0.975)^*^	91.8	87.3	0.791	0.507**
FAS	0.890^a^	0.885 (0.855,0.909)	0.950 (0.913,0.975)^*^	91.8	87.3	0.791	0.508**
EKFC	0.890^a^	0.862 (0.829,0.888)	0.951 (0.914,0.975)^*^	92.4	87.3	0.797	0.528**
Diabetic (*N* = 293)							
CKD-EPI	0.869^a^	0.851 (0.819,0.878)	0.934 (0.900,0.960)^*^	84.7	90.8	0.754	0.484**
LMR	0.869^a^	0.858 (0.825,0.885)	0.934 (0.899,0.959)^*^	87.3	86.2	0.734	0.462**
BIS1	0.869^a^	0.866 (0.835,0.891)	0.934 (0.900,0.960)^*^	84.7	90.8	0.754	0.466**
FAS	0.872^a^	0.872 (0.841,0.897)	0.935 (0.900,0.960)^*^	83.3	90.8	0.741	0.478**
EKFC	0.871^a^	0.858 (0.825,0.884)	0.935 (0.901,0.961)^*^	87.7	86.2	0.739	0.477**
Non-diabetic(*N* = 319)							
CKD-EPI	0.871^a^	0.834 (0.801,0.862)	0.919 (0.884,0.947)^*^	85.8	86.0	0.719	0.480**
LMR	0.872^a^	0.850 (0.818,0.877)	0.920 (0.885,0.947)^*^	87.6	83.9	0.715	0.481**
BIS1	0.867^a^	0.859 (0.828,0.885)	0.918 (0.882,0.946)^*^	88.1	82.8	0.709	0.500**
FAS	0.870^a^	0.856 (0.824,0.883)	0.920 (0.884,0.947)^*^	87.2	86.0	0.732	0.484**
EKFC	0.872^a^	0.841 (0.808,0.869)	0.919 (0.884,0.947)^*^	88.9	83.9	0.728	0.474**

The CCC ranges between −1 and 1; 1 denotes perfect agreement, greater than 0.990, almost perfect agreement; 0.950 to 0.990, substantial agreement; 0.900 to 0.949, moderate agreement; and less than 0.900, poor agreement. Kappa value 0.21–0.40 is considered mild agreement, 0.41–0.60 moderate agreement, 0.61–0.80 substantial agreement, and 0.81–1.00 near perfect agreement

*Abbreviations*: *AUC* Area under the receiver operating characteristic curve, *CCC* Concordance correlation coefficient; *95%CI* 95% confidence interval. *CKD-EPI* Chronic kidney disease epidemiology collaboration, *LMR* Lund-Malmö Revised, *BIS1* Berlin Initiative Study 1, *FAS* Full age spectrum equation, *EKFC* European Kidney Function Consortium, *EKFC* European Kidney Function Consortium. ^a^ *P* < 0.01, * *P* < 0.05, ** *P* < 0.001 compared with mGFR

**Table 4:** Detailed performance of the five GFR-Estimating Equations

	Bias	Precision	Accuracy		MAE	GFR category Misclassification(%)
	Median difference	IQR	P30(%)	RMSE		
All Participants(*N* = 612)						
CKD-EPI	0.795^a^	16.2	64.9	14.5	10.7	37.4
LMR	−3.015^a^	13.4	69.9*	13.3	9.79	37.4
BIS1	−0.050	12.7	73.9**	12.9	9.26	35.3
FAS	−1.765^a^	13.1	72.4**	12.1	9.48	36.3
EKFC	−1.970^a^	13.5	69.0*	13.3	9.71	36.9
Participants Aged 65–79 Years(*N* = 444)						
CKD-EPI	0.290	15.3	65.1	14.2	10.40	38.1
LMR	−3.140^a^	12.7	69.1*	13.5	9.80	38.3
BIS1	0.150	12.7	73.0**	13.1	9.33	36.5
FAS	−1.670^a^	12.7	72.1**	13.2	9.46	37.2
EKFC	−2.180^a^	13.0	68.9*	13.5	9.68	37.4
Participants Aged ≥80 Years(*N* = 168)						
CKD-EPI	3.135^a^	18.0	64.3	15.1	11.30	35.7
LMR	−2.615^a^	14.2	72.0*	12.9	9.76	35.1
BIS1	−1.095	13.8	76.2*	12.1	9.11	32.1
FAS	−1.810	13.5	73.2*	12.7	9.54	33.9
EKFC	−1.800^a^	14.8	69.0	13.0	9.82	35.7
Male(*N* = 386)						
CKD-EPI	0.665	15.7	68.4	14.2	10.34	36.0
LMR	−3.890^a^	14.1	73.3*	13.5	9.91	39.4
BIS1	−0.770	14.0	75.1*	12.8	9.37	35.2
FAS	−1.810^a^	13.5	75.1	12.9	9.42	36.5
EKFC	−2.145^a^	13.6	71.5*	13.4	9.73	38.3
Female(*N* = 226)						
CKD-EPI	1.260^a^	18.0	58.8	15.0	10.37	39.8
LMR	−1.950	13.7	64.2	13.1	9.93	34.1
BIS1	0.370^a^	13.1	71.7**	12.9	9.39	35.4
FAS	−1.590	12.8	67.7*	13.4	9.45	35.8
EKFC	−1.815^a^	14.0	64.6*	13.3	9.76	34.5
mGFR≥60 ml/min/1.73m^2^(*N* = 158)						
CKD-EPI	1.590	23.8	80.4	17.6	13.96	39.9
LMR	−7.860^a^	20.5	81.6	18.9	14.37	44.9
BIS1	−8.285^a^	19.9	79.7	18.8	14.57	47.5
FAS	−6.480^a^	21.4	77.2	18.9	14.60	46.8
EKFC	−5.760^a^	21.7	81.6	18.3	13.81	44.9
mGFR<60 ml/min/1.73m^2^(*N* = 454)						
CKD-EPI	0.720^a^	15.1	59.5	13.2	9.50	36.6
LMR	−2.065^a^	12.3	65.9*	10.8	8.20	34.8
BIS1	1.220^a^	11.9	71.8**	10.0	7.42	31.1
FAS	−0.640	11.7	70.7**	10.3	7.70	32.6
EKFC	−1.120^a^	13.1	64.5**	11.1	8.29	34.1
Diabetic (*N* = 293)						
CKD-EPI	0.730	16.3	65.5	13.6	10.15	36.9
LMR	−3.150^a^	12.9	69.6	12.7	9.36	37.9
BIS1	−0.370	12.8	74.1*	11.9	8.71	35.8
FAS	−1.820^a^	12.3	71.7*	12.0	8.83	36.2
EKFC	−1.990^a^	12.5	70.0*	12.5	9.17	36.5
Non-diabetic(*N* = 319)						
CKD-EPI	0.960^a^	16.7	64.3	15.2	11.1	37.9
LMR	−2.830^a^	14.0	70.2*	14.0	10.2	37.0
BIS1	0.110	13.0	73.7**	13.7	9.8	34.8
FAS	−1.760	13.9	73.0**	14.0	10.1	36.4
EKFC	−1.950^a^	14.7	68.0*	14.0	10.2	37.3

Median difference in bias is the difference between equation biases (estimated GFR minus measured GFR); *P30* Percentage of estimates within 30% of the measured value, *IQR* The inter-quartile range of difference, *RMSE* Root mean square error, *MAE* Mean absolute error, *CKD-EPI* Chronic kidney disease epidemiology collaboration, *LMR* Lund-Malmö Revised, *BIS1* Berlin Initiative Study 1, *FAS* Full age spectrum equation, *EKFC* European Kidney Function Consortium

^a^*P* < 0.05, compared with mGFR; ^*^ *P* < 0.05, **P < 0.001,compared with CKD-EPI

### Performance of different equations in different subgroups

In the age subgroup, as based on Rs, CCC, ROC^AUC^ and Cohen’s kappa, the five equations performed better in the 65–79 year-old age group than in the ≥80 year-old age group **(**Table 3)**.** In the 65–79 year-old group, the CKD-EPI equation was unbiased (bias: 0.290, *P* = 0.213), as was the BIS1 equation (bias: 0.150, *P* = 0.456). In the ≥80 year-old age group, all equations underestimated GFR, except for the CKD-EPI equation (bias: 3.135, *P*<0.001) and the BIS1 equation was also unbiased (bias: -1.095, *P* = 0.318) (Table 4). Concerning precision, the IQR of the FAS and BIS1 equations in the two age subgroups did not differ obviously but were smaller than other equations. The BIS1 equation showed the highest P30 (73.0 and 76.2%), followed by FAS (72.1 and 73.2%) in the two age subgroups (the 65–79 years group for the former and the ≥80 years group for the latter). Additionally, the BIS1 equation exhibited the lowest RMSE and GFR category misclassification in these two age subgroups. In contrast, the CKD-EPI equation performed the worst in P30, RMSE, and GFR category misclassification in the two age subgroups. In general, the five equations were as accurate in the 65–79 years group as in the ≥80 years group.

Comparing between two sex subgroups showed the five equations had a better diagnostic performance in the female group. In the male group, all the equations underestimated GFR, except for the CKD-EPI equation (bias: 0.665, *P* = 0.067), which was unbiased. The FAS equation (bias: -1.590, *P* = 0.598) and LMR equation (bias: -1.950, *P* = 0.219) were unbiased in the female group. As with the age subgroups, the BIS1 equation showed the smallest IQR and MAE, the highest P30, and the lowest misclassification rate in the sex subgroups. Overall, the accuracy of the five equations was similar between the sexes (Table 4).

In the subgroup with mGFR≥60 ml/min/1.73 m^2^, the reported bias, IQR, RMSE, and MAE of all equations were generally higher than in the subgroup with mGFR<60 ml/min/1.73 m^2^. Although the P30 of the five equations in the mGFR≥60 ml/min/1.73 m^2^ subgroup was close to 80%, the P30 of the equations was not significantly different compared to the CKD-EPI equation. Bias was significantly high in the mGFR≥60 ml/min/1.73 m^2^ subgroup, except for the CKD-EPI equation. None of the 5 equations performed ideally in the subgroup with mGFR≥60 ml/min/1.73 m^2^. Thus, all equations had higher accuracy in the mGFR<60 ml/min/1.73 m^2^ subgroup. For the mGFR< 60 ml/min/1.73 m^2^ subgroup, the CKD-EPI and BIS1 equations overestimated GFR, and the FAS equation (bias: -0.640, *P* = 0.737) was unbiased. The BIS1 equation displayed a relatively lower IQR (11.9 ml/min/1.73 m^2^), the greatest accuracy (P30 reached 71.8%, RMSE was 10.0), and the lowest GFR category misclassification (31.1%). The FAS equation performed slightly inferior to the BIS1 equation in the mGFR< 60 ml/min/1.73 m^2^ subgroup. The performance of the CKD-EPI equation was worse than that of the four equations in the mGFR< 60 ml/min/1.73 m^2^ subgroup (Table 4).

In contrast, the diagnostic performance of the 5 equations did not differ distinctly between diabetic and nondiabetic subgroups. The CKD-EPI equation (bias: 0.730, *P* = 0.085) and BIS1 equation (bias: -0.370, *P* = 0.640) were unbiased and the BIS1 equation showed the highest accuracy (P30: 74. 1%; RMSE: 8.71) and the lowest GFR category misclassification (35.8%) in the diabetic subgroup. Similarly, the unbiased BIS1 equation (bias: 0.110, *P* = 0.696) was the most accurate (P30: 73.7%; RMSE: 9.8) and had the lowest GFR category misclassification (34.8%) in the nondiabetic subgroup. None of the five equations performed notably better in either subgroup.

## Discussion

There was no unanimous conclusion about which equation was more suitable for Chinese elderly individuals before. In this significant clinical study, which analysed the applicability of the newly developed equation in Chinese elderly inpatients and compared it with equations recommended by guidelines and developed in the elderly population, the performance of the EKFC equation was not better than previous equations in patients older than 65 years. Regarding P30, none of the equations reached the 90% recommended by the guidelines [13], and diagnostic performance was similar among five equations in different subgroups in terms of the correlation coefficient, concordance correlation coefficient and ROC^AUC^. Comparing between subgroups showed the diagnostic value of the five equations was worse in the ≥80 years and male subgroups. The accuracy of the five equations was similar between the 65–79 years and ≥ 80 years subgroups, male and female subgroups, and diabetic and nondiabetic subgroups, with BIS1 being the best performer. Nevertheless, all equations had higher accuracy in the mGFR<60 ml/min/1.73 m^2^ than the mGFR≥60 ml/min/1.73 m^2^ subgroup. Overall, the BIS1 equation displayed a superior performance in Chinese elderly individuals with moderate to severe renal impairment.

Because GFR has great influence on the diagnosis and medical treatment of elderly individuals who require drug dosage adjustment and elderly individuals experience physiological changes in renal function, such as increases in the numbers of sclerosing glomeruli, renal cortical atrophy, interstitial fibrosis, and other structural changes [14], it is necessary to measure GFR accurately in these individuals, especially those with impaired kidney function. According to a survey among 2974 expected living kidney donors from 18 centres in the UK, the mGFR of people before the age of 35 was approximately 100 ml/min/1.73 m^2^, of men over 65 years old was approximately 80 ml/min/1.73 m^2^ and of women over 65 years old was approximately 75 ml/min/1.73 m^2^, indicating that GFR decreases linearly with age [15]. Pottel et al. also reported a similar relationship between age and GFR [16]. Therefore, we should pay more attention to age-related GFR changes. Clinically, SCR is the most commonly used biomarker to assess renal function, but it is affected by muscle mass and diet, especially in elderly individuals [17]. Thus, is remains unclear whether the SCR-based equations developed in the nonelderly population can accurately predict renal function in the elderly.

Although each equation showed good diagnostic efficiency in similar populations, each had limitations [17]. The CKD-EPI equation was not developed for elderly individuals, with only 13.0% of individuals 65 years or older in the data sets [6]. Therefore, it is foreseeable that the utility of the equation is limited in the elderly. With regard to bias, the CKD-EPI equation was generally better than other equations but performed worse than BIS1 in this study. However, P30 became especially important when compared with bias in the accurate evaluation of equations. In this study, the CKD-EPI equation performed the worst in terms of precision, accuracy and GFR category misclassification in the whole cohort. In a study involving elderly individuals ≥65 years old, the CKD-EPI equation was also found to not be optimal when compared to the BIS1, FAS, and LMR equations [11].

The Lund-Malmo equations developed based on Swedish Caucasians and revised later included 51% of the population over 60 years and showed higher accuracy compared to the CKD-EPI equation [18]. In the present study, the LMR equation was less accurate than the BIS1, FAS, and EKFC equations for calculating GFR. A study involving diabetic patients showed the LMR equation reportedly performed equally well when compared to CKD-EPI [19], and a multicentre analysis of older people in Europe showed the same results [20]. Similarly, in the study, the LMR equation was not significantly better than CKD-EPI equation in the diabetic subgroup.

The BIS1 equation was developed in elderly individuals aged 70 years or above (average age 78.3 years), with reasonable performance in terms of age. A previous study found that although the SCR level of the elderly fluctuated greatly, the BIS1 equation performed better than the CKD-EPI equation in terms of bias (0. 11 vs. 8.98), IQR (11.14 vs. 13. 04), and P30 (95.10% vs. 77.90%) [9]. In this study, BIS1 also had better performance than the other equations. A study of Caucasian subjects concluded that compared with CKD-EPI, BIS1 was most suitable for the elderly, especially patients at CKD stages 1 to 3 [21]. Other studies in Chinese elderly individuals have found that the BIS1 equation exhibited relatively good performance, especially in those with GFR < 60 ml/min/1.73 m^2^ [22, 23], similar to the present study. Therefore, the BIS1 equation seems to be a good tool for estimating GFR in elderly individuals. This study showed that BIS1 performed better, but other studies have reported conflicting results [11, 20, 24, 25]. Hence, whether the BIS1 equation can be applied across ethnicities requires more analysis.

To avoid discontinuity in age, Pottel developed the FAS equation in 2016, which was the first to cover all ages. The principle was to use age-normalized SCR, corresponding to the age/sex of healthy people. Pottel showed that compared with the CKD-EPI equation, the FAS equation had less bias (5.6 for the former and − 1.1 for the latter) and higher accuracy (P30: 77.6 and 86.1%, respectively), slightly better than the BIS1 equation, in 1764 patients aged 70 years and older, with a mean [SD] mGFR of 55.7 [20.6] ml/min/1.73 m^2^ [10, 26]. In the community-dwelling elderly Icelandic Age, Gene/Environment Susceptibility-Kidney (AGES-Kidney) study involving those with an age range of 74–91 years and a mean mGFR of 64.0 ml/min/1.73 m^2^, the FAS equation performed relatively better than the CKD-EPI equation in terms of P30 (95.8 vs. 91.7%), but the bias of FAS was higher (− 5.7 vs. 2.7, 27]. The results of the AGES-Kidney study [27] and Chen’s study [23] were similar to those of this study in terms of CKD-EPI and FAS. Hence, the FAS equation was comparatively suitable for determining GFR in the elderly, especially for those with a lower GFR. Bland–Altman plots in this study and Peng’s study also illustrated that the GFR calculated by the FAS equation has a good correlation (mean − 1.3 and − 1.2, respectively) with mGFR determined by the dual plasma sample clearance method [28]. However, the FAS equation was inferior to the BIS1 equation in the present study.

The EKFC equation was developed in a large cohort of patients referred for GFR measurement in a European population (all non-black) in 2020. In that population, the mean age (SD) was 42.4 (25.2) years, and the mean (SD) mGFR was 76.9 (32.2) ml/min/1.73 m^2^. The age-related coefficient of this equation was adjusted to 0.990, and different exponential coefficients were applied according to the SCR level, such as in the CKD-EPI (not FAS) equation. That study showed that the EKFC equation was better than the CKD-EPI and FAS equations (bias, IQR, and P30) in the elderly group [12]. Nonetheless, it had been shown that compared with the CKD-EPI equation, the EKFC equation had a similar bias in homozygous populations, but with a smaller P30 and worse performance in non-Caucasians of any age [29]. This study further illustrated that EKFC was not significantly better than CKD-EPI and slightly worse than FAS and BIS1 for Chinese elderly individuals.

Chronic kidney disease (CKD) is usually asymptomatic in its early stages, which means that people are not identified or treated until the late stage of the disease. Improving early recognition and diagnosis of CKD can effectively reduce mortality and complications. Although SCR is the most commonly used biomarker for estimating GFR, this method has many limitations. Cystatin C (CysC) is a small-molecular-weight cystine protease inhibitor that can be produced by all nucleated cells [30]. In the assessment of renal function, CysC is less affected by muscle mass and independent of age and sex [31–34]. Furthermore, CysC is more sensitive and specific than SCR and can reflect mild damage to renal function and is thus an alternative and good biomarker of kidney function in elderly individuals with reduced muscle mass [35, 36]. Other studies have shown that CysC is also more accurate than SCR in predicting the mortality risk of elderly patients with CKD [37, 38]. Regardless, the use of the CysC-based equation might be less suitable than the SCR-based equation [39]. This might be because the number of people diagnosed with CKD decreases but more patients are classified as having advanced CKD which might bring higher costs [39]. Therefore, when the condition of a patient is stable, such as showing a small weight fluctuation, an SCR-based equation may be considered to assess renal function [17], as in this study.

One of the strengths of this study was that it is the first clinical study to use the EKFC equation to evaluate the glomerular filtration rate in elderly Chinese inpatients and employed the Tc-99 m-DTPA dual plasma sample clearance method as a reference, which was related to the clearance of inulin and reproducible [40].

### Limitations of the study

There were several limitations to this study. First, this was a single-centre study of 612 elderly inpatients, and more thematic multicentre studies should be conducted in China. Second, mGFR in this study was obtained via Tc-99 m-DTPA, though this was different from the plasma clearance rate of exogenous filter markers used in other data sets employed to develop equations. Therefore, changes in the measured value of mGFR may partially affect the effective value. Gold standards based on different measures may also cause deviations. Third, this study only measured the serum value once in each subject, which may affect the accuracy of the results.

## Conclusion

The data from the present study indicated that among patients aged 65 years and older with GFR estimated by CDK-EPI, LMR, BIS1, FAS, and EKFC, the new creatinine-based EKFC equation did not show superior diagnostic performance and accuracy. Indeed, the BIS1 equation might be the most accurate for estimating GFR in Chinese individuals 65 years and older with moderate to severe renal impairment.

## Data Availability

The dataset supporting the conclusions of this article is included within the. article. The datasets generated and/or analysed during the current study are available from the corresponding author upon reasonable request.

## Abbreviations

SCR: Serum creatinine
BSA: Body surface area
Bun: Blood urea nitrogen
Alb: Albumin
mGFR: Measured glomerular filtration rate
eGFR: Estimated glomerular filtration rate
CKD-EPI: Chronic kidney disease epidemiology collaboration
LMR: Lund-Malmö Revised
FAS: Full age spectrum equation
BIS1: Berlin Initiative Study 1
EKFC: European Kidney Function Consortium
AUC: Area under the receiver operating characteristic curve
CCC: Concordance correlation
P30: Percentage of estimates within 30% of the measured value
IQR: The inter-quartile range of difference
RMSE: Root mean square error
MAE: Mean absolute error
